# Laparoscopic Adrenalectomy for Adrenal Tumors

**DOI:** 10.1155/2014/241854

**Published:** 2014-07-15

**Authors:** Sun Chuan-yu, Ho Yat-faat, Ding Wei-hong, Gou Yuan-cheng, Hu Qing-feng, Xu Ke, Gu Bin, Xia Guo-wei

**Affiliations:** Department of Urology, Huashan Hospital of Fudan University, Shanghai 200040, China

## Abstract

*Objective*. To evaluate the indication and the clinical value of laparoscopic adrenalectomy of different types of adrenal tumor. *Methods*. From 2009 to 2014, a total of 110 patients were diagnosed with adrenal benign tumor by CT scan and we performed laparoscopic adrenalectomy. The laparoscopic approach has been the procedure of choice for surgery of benign adrenal tumors, and the upper limit of tumor size was thought to be 6 cm. *Results*. 109 of 110 cases were successful; only one was converted to open surgery due to bleeding. The average operating time and intraoperative blood loss of pheochromocytoma were significantly more than the benign tumors (*P* < 0.05). After 3 months of follow-up, the preoperative symptoms were relieved and there was no recurrence. *Conclusions*. Laparoscopic adrenalectomy has the advantages of minimal invasion, less blood loss, fewer complications, quicker recovery, and shorter hospital stay. The full preparation before operation can decrease the average operating time and intraoperative blood loss of pheochromocytomas. Laparoscopic adrenalectomy should be considered as the first choice treatment for the resection of adrenal benign tumor.

## 1. Introduction

Adrenalectomy is the standard treatment for adrenal gland disorders such as secretory tumors [1]. Laparoscopic procedures have been proposed to reproduce the surgical steps of open surgery and decrease morbidity, postoperative pain, and hospital stay. Laparoscopic surgery for adrenal tumors was firstly reported in 1992 by Gagner et al. who used the transperitoneal approach in three patients [2]. The benefits of laparoscopy on postoperative pain, cosmesis, hospital stay, and convalescence are widely recognized. Current efforts are aimed at further reducing the morbidity associated with minimally invasive surgery [3]. The upper limit of tumor size for laparoscopic surgery was thought to be 6 cm, for two main reasons: (i) the technique to remove a large tumor by laparoscopy is difficult; (ii) large tumors have higher possibility of malignancy [4].

But, there are some series of reports that laparoscopic surgery of the large pheochromocytomas was safe and effective. From 2009 to 2014, we evaluated 110 cases of adrenal diseases including the diameter of >5 cm adrenal tumor who underwent laparoscopic adrenalectomy.

## 2. Surgical Procedure

All patients were treated by retroperitoneal laparoscopic adrenalectomy. The procedures are usually performed with the patient in a flank position (90°) with the operating table fixed and then we elevated and exposed the surgical area between the costal margin and the iliac crest, and a 2 cm skin incision was made on the middle axillary line above the iliac crest. The fascia was exposed and incised over the same length, the muscle layers were divided bluntly, and the peritoneum was free from the abdominal wall by a finger dissection. Inserting the 10 mm trochar, CO_2_ was insufflated to a maximum pressure of 2.0 kPa, and then the laparoscope was introduced. The second trochar was placed on the posterior axillary line under the costal arch. The peritoneal sac was mobilized medially to allow for the introduction of two additional trochars on the anterior axillary line at the level of the two preceding trochars. Gerota's fascia was identified and opened to expose the upper pole of the kidney and the adrenal gland.

## 3. Statistical Analysis and Patients

Statistical analysis used two-sample Wilcoxon rank-sum (Mann-Whitney) test via Stata7 software. *P* value less than 0.05 was considered statistically significant.

From 2009 to 2014, 110 patients diagnosed with adrenal benign tumors by CT scan were treated by retroperitoneal laparoscopic adrenalectomy at Huashan Hospital of Fudan University, China. The patients were analyzed retrospectively, including 65 on the left, 39 on the right side, and 6 on both sides, in 43 men and 67 women aged 22–79 (mean 50) years. The laparoscopic approach has been the procedure of choice for surgery of benign adrenal tumors, and the upper limit of tumor size was thought to be 6 cm.

## 4. Results

109 cases were successfully completed, but one case was converted into open surgery because the bleeding was out of control (pathology diagnosed adrenal cortical carcinoma). The mean operating time and intraoperative blood loss were 102.536 (35–327) mins and 81.454 (5–700) mL, respectively. Postoperative complications developed in 3 cases. One case developed effusion above kidney, and one case developed pneumonia. The last case was infected by MRSA. The postoperative hospitalization times were 6.1636 (2–30) days.

**Figure 1:**
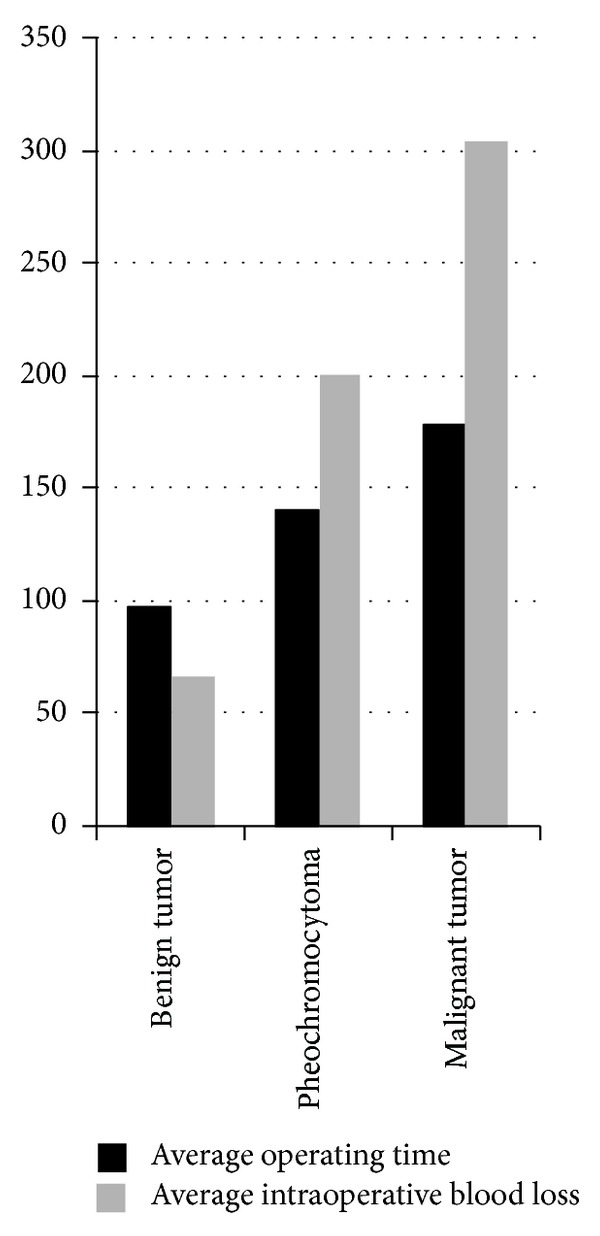
The average operating time and average intraoperative blood loss of different types of tumors. The intraoperative blood loss and average operating time of pheochromocytomas were significantly more than the benign tumors (*P* < 0.05), but malignant tumors between benign tumors or pheochromocytomas were without statistical significance (*P* > 0.05).

Cortical adenoma was the most common pathology (62.727%), followed by cortical hyperplasia and myelolipoma (9.091%), pheochromocytoma (6.364%), cyst (3.636%), adrenal cortical carcinoma (2.727%), lipoma (1.818%), neurofibroma (0.909%), spindle cell tumor (0.909%), cystic tumor (0.909%), inflammatory mass (0.909%), and lymphangioma (0.909%) (Table 2). Most complaints of the patients were symptomless (Table 1). We divided all patients into 3 groups which were benign tumor, pheochromocytoma, and malignant tumor groups and analyzed the average diameter, operating time, and intraoperative blood loss of each group. Based on pathologic specimens and CT scans, the tumor average diameter was 2.688 cm (0.6–6). The average diameters of benign tumors, pheochromocytomas, and malignant tumors were 2.607 cm (0.6–6), 3.214 cm (2–3.5), and 3.5 cm (3-4), respectively. The malignant tumors were bigger than the other tumors but without statistical significance (*P* > 0.05). The average operating time was 102.536 mins (35–327). One case was converted into open surgery and the operating time was 327 mins because the bleeding was out of control (pathology diagnosed adrenal cortical carcinoma). The average operating times of benign tumors, pheochromocytomas, and malignant tumors were 97.62 mins (35–280), 140.14 mins (90–205), and 178.67 mins (60–327), respectively. The intraoperative blood loss of benign tumors, pheochromocytomas, and malignant tumors was 8 mL (8–400), 85.71 (0–400), and 0 mL, respectively. The intraoperative blood loss and average operating time of pheochromocytomas were significantly more than the benign tumors (*P* < 0.05), but malignant tumors between benign tumors or pheochromocytomas were without statistical significance (*P* > 0.05) (Figure 1).

## 5. Conclusion

Laparoscopic adrenalectomy includes transperitoneal approach and retroperitoneal approach. The choice of the surgical approaches of laparoscopic adrenalectomy depends on the operators' habits and experience. We prefer retroperitoneal approach, which is not disturbed by abdominal organs. The intraoperative blood loss of pheochromocytomas was significantly more than the benign tumors (*P* < 0.05). We recommend the following to prevent intraoperative blood loss and decrease operation time. First, control the blood pressure before operation by Cardura. If it is necessary, use *β* receptor blocker. The full complement of crystalloid and colloid expands fluid volume. Second, the operators should carefully read the CT and MRI imaging data and ensure the location of the tumor and the relationship with the surrounding organs and tissues. Third, during the operation, clamp the fat around adrenal gland gently and cut off the small blood vessel by ultrasonic knife to decrease bleeding to maintain visual field. The vital management is to dissociate adrenal central vein enough, with 3 titanium clips ligation. If the vessel is thick, using the Ham-o-Lock ligation is necessary. With regard to the diameter >5 cm of adrenal tumor or large pheochromocytomas, we prefer transabdominal approach.

Compared to retroperitoneal LA, transabdominal approach is risky and can injure intestine, liver, spleen, and other adjacent organs but has the advantages of the obvious anatomical landmarks, wider operation space, and more convenience during the operation.

The large adrenal tumors have higher malignant incident rate, and most of the researchers do not recommend resecting the malignant tumors by laparoscopic adrenalectomy because those are large, rich in blood supply, with thin envelope surrounding tissue invasion. In addition, laparoscopic clamping separation may lead to tumor rupture, local recurrence, or trochar implant. Some researchers held negative attitude of laparoscopic operation for larger adrenal tumors. Nevertheless, confirming the adrenal malignant tumors is difficult by imaging. Under imaging, the malignant tumors present irregular boundary, local invasion, and uneven and high density, and so do benign tumors [5]. Sturgeon et al. discovered more malignant incidence rate of the large adrenal tumor (<4 cm = 5%, ≧4 cm = 10%, and ≧8 cm = 47%) [6]. Our data showed three malignant tumors were 3 cm, 3.5 cm, and 4 cm, respectively (<4 cm = 2.22%, ≧4 cm = 5%). If we only consider the tumor size as the surgical approaching standard, many patients will undergo unnecessary open operation. Kebebew et al. and Lombardi et al showed 3-year disease-free survival rate and local recurrence rate were similar to open operation. As for trocar metastasis, it is a rare complication of laparoscopic operation [7, 8].

Our data showed that the average diameter, operating time, and intraoperative blood loss of malignant tumors were not significantly different to pheochromocytomas and benign tumors (*P* > 0.05) indicating that the laparoscopic operation of adrenal malignant tumor is safe. But for those tumors invading surrounding tissue and blood vessels, they are difficult to control and separate, when patients have serious bleeding circumstances, we should seize the opportunity to convert into open operation. In view of this, implementing laparoscopic operation of adrenal malignant tumors is not the contraindication. But before being widely carried out, multicenter prospective controlled trials on laparoscopic and open radical adrenalectomy of malignant tumors are necessary.

## Figures and Tables

**Table 1:** The complaints of all patients.

Complaint	
Hypertension	25
Lumbar/abdominal pain	9
Limb weakness	7
Limb weakness and hypertension	4
Cushing syndrome	6
Limb numbness	4
Hyperglycemia	1
Symptomless	54

**Table 2:** The number, characteristics, and probability of pathology.

Pathology	Number	Characteristics	Probability
Cortical adenomas	69	Golden yellow	62.727
Pheochromocytoma	7	Grey yellow and red	6.364
Cortical hyperplasia	10	Grey yellow	9.091
Neurofibroma	1	Milky white	0.909
Myelolipoma	10	Grey yellow and red	9.091
Cortical carcinoma	3	Golden yellow or grey red	2.727
The spindle cell tumor	1	Yellow and white	0.909
Cyst	4	Cystic	3.636
Cystic tumor	1	Cystic	0.909
Inflammatory mass	1	Grey white	0.909
Lipoma	2	Grey yellow	1.818
lymphangioma	1	Cystic	0.909

The probability is accurate to 3 digits after the decimal point.
